# Preparation, Structure, and Properties of Silk Fabric Grafted with 2-Hydroxypropyl Methacrylate Using the HRP Biocatalyzed ATRP Method

**DOI:** 10.3390/polym10050557

**Published:** 2018-05-21

**Authors:** Jinqiu Yang, Shenzhou Lu, Tieling Xing, Guoqiang Chen

**Affiliations:** National Engineering Laboratory for Modern Silk, Soochow University, Suzhou 215123, China; yjq8440@163.com (J.Y.); lushenzhou@suda.edu.cn (S.L.); chenguojiang@suda.edu.cn (G.C.)

**Keywords:** horseradish peroxidase, atom transfer radical polymerization, bio-catalysis, silk

## Abstract

Atom transfer radical polymerization (ATRP) is a “living”/controlled radical polymerization, which is also used for surface grafting of various materials including textiles. However, the commonly used metal complex catalyst, CuBr, is mildly toxic and results in unwanted color for textiles. In order to replace the transition metal catalyst of surface-initiated ATRP, the possibility of HRP biocatalyst was investigated in this work. 2-hydroxypropyl methacrylate (HPMA) was grafted onto the surface of silk fabric using the horseradish peroxidase (HRP) biocatalyzed ATRP method, which is used to improve the crease resistance of silk fabric. The structure of grafted silk fabric was characterized by Fourier transform infrared spectrum, X-ray photoelectron spectroscopy, thermogravimetic analysis, and scanning electron microscopy. The results showed that HPMA was successfully grafted onto silk fabric. Compared with the control silk sample, the wrinkle recovery property of grafted silk fabric was greatly improved, especially the wet crease recovery property. However, the whiteness, breaking strength, and moisture regain of grafted silk fabric decreased somewhat. The present work provides a novel, biocatalyzed, environmentally friendly ATRP method to obtain functional silk fabric, which is favorable for clothing application and has potential for medical materials.

## 1. Introduction

Silk is a natural protein fiber. It is widely used in the textile field because of its outstanding mechanical strength, wear comfortability, and elegant luster, which has been praised as the queen of fibers [1,2,3]. However, silk has some shortcomings, such as bad crease recovery property and photo-yellowing stability [4,5,6]. Silk is very easy to wrinkle during wearing, especially after sweating or washing. This shortage seriously affects the utilization of silk and limits its application scope. In recently years, high-quality and functional silk has be prepared to improve the performance of the end products and satisfy the specific requirements with vinyl monomers using the grafting technique [7,8]. Silk fabric can be grafted with vinyl monomer by conventional radical polymerization through various chemical initiators or by irradiation, and the controlled/living radical polymerization process (CRP). Atom transfer free radical polymerization (ATRP) is the most widely used CRP method [9].The ATRP method is used in mild reaction conditions, which provide well-defined polymers and show high tolerance for monomer structures with a variety of functional groups [10,11,12,13]. Surface-initiated ATRP has been applied in materials to provide their wettability, wrinkle resistance, anti-bacterial property, and flame retardancy, etc. Teramoto [14] prepared cellulose diacetate-graft-poly(lactic acid)s (CDA-g-PLAs) through ATRP, and the thermal characteristics of cellulose diacetatewaereimproved. Kang [15] synthesized ethyl cellulose-graft-poly(2-hydroxyethyl methacrylate) (EC-graft-PHEMA) copolymers using the ATRP in methanol to improve the hydrophilic property of ethyl cellulose. Xing [16] prepared multi-functional silk with flame retardancy and antibacterial properties using flame retardant dimethyl methacryloyloxyethyl phosphate (DMMEP) as the first monomer and dimethylaminoethyl methacrylate (DMAEMA) as the second monomer using the ATRP method.

However, a major disadvantage of the ATRP method is the usage of the transition metal complex catalyst, which is usually used inrelatively large amounts. The commonly used metal catalyst, CuBr, is mildly toxic and renders ATRP environmentally unfavorable. The copper ion residuals are difficult to remove from the polymer materials and hinder the application of the resulting polymers in biomedical and food fields [17,18]. Meanwhile, the colored catalyst is easy to stain the grafted materials and results in the unwanted color for textiles.

Enzymes can be an alternative to the transition metal catalyst because of their non-toxicity and eco-friendly nature [8,19]. A lot of enzymes have catalytic active sites comprising metal ions like peroxidase. Horseradish peroxidase (HRP; EC1.11.1.7) is a kind of heme protein, containing active sites on the ferric protoporphyrin ring. Recently, HRP-catalyzed ATRP has been used for the synthesis of polymers. Sigget al. [20] catalyzed N-isopropyl-acrylamide using activators generated by electron transfer for the ATRP (ARGET ATRP) method using HRP. This method allows an ATRP process to be conducted with a tiny amount of transition metal catalyst in thepresence of excess reducing agent such as ascorbic acid, which could effectively scavenge and remove dissolved oxygen from the polymerization system. Renggli [21] obtained a protein cage nano reactor using HRP as biocatalyst, which was further polymerized with an acrylate. These works provided the foundation for HRPcatalyzed grafting using the surface-initiated ATRP method.

**Figure 1:**
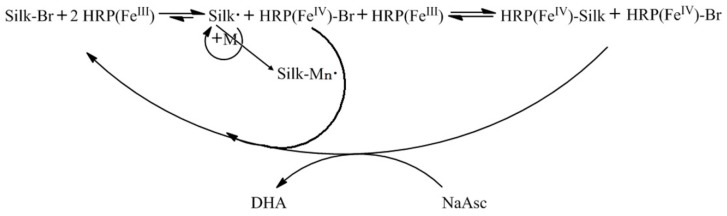
Schematic of silk grafted with monomers using HRP biocatalytic ATRP (M = vinyl monomer, M_n_ = polymer with n repeat units, NaAsc = sodium ascorbate, DHA = dehydroascorbic acid).

The active group of HRP contains a ferric protoporphyrin structure to catalyze ATRP, which is equivalent to the CuBr/ligand metal complex catalyst. The schematic of silk fabric grafted with monomers using the HRP-catalyzed ATRP method is shown in Figure 1.

In this work, HRP was used as biocatalyst for the ATRP grafting of 2-hydroxypropyl methacrylate (HPMA) on silk surface to improve the wrinkle resistance of silk fabric. Sodiumascorbate (NaAsc) with higher water solubility than ascorbic acid was used as the reducing agent in ARGET ATRP. The structure and properties of the grafted silk fabric were investigated.

## 2. Materials and Methods

### 2.1. Materials and Reagents

Degummed silk fabric, with a 36 g/m^2^ density, was purchased from Suzhou Huajia Silk Group. 2-Hydroxypropyl methacrylate (HPMA) was purchased from Macklin. Triethylamine (TEA) and tetrahydrofuran (THF) were distilled under reduced pressure before use. Horseradish peroxidase (HRP; EC1.11.1.7) was supplied by Aladdin Reagent and stored at −20 °C. All other reagents in this study were used without further purification.

### 2.2. Preparation of the Silk Macroinitiator

The silk fabric (1 g) reacted with 2-bromoisobutyryl bromide (BriB-Br) (2.22 mL) in the presence ofTEA (1.23 mL) and 4-(dimethylamino) pyridine (DMAP, 0.5 g). The mixture was stirred at 10 °C for 1 h, then warmed up to 50 °C for 24 h. The silk sample was thoroughly washed with water and finally dried at 60 °C in vacuum oven [10,15]. Thus, the silk-Br macroinitiator was prepared.

### 2.3. Surface-Initiated ATRP

#### 2.3.1. HRP-Mediated Grafting of HPMA on Silk Fabric’s Surfaces

The silk-Br macroinitiator (1 g) was incubated with 50 mL of phosphate buffer (1/15 M, pH 8.0), containing a certain volume of monomer (HPMA), l-sodiumascorbate (NaAsc), and HRP in a 100 mL round-bottom flask([HPMA] = 0.16 mol/L, [HRP] = 0.027 mmol/L, n(HRP):n(NaAsc) = 1:150). Then, the flask was evacuated and filled with nitrogen for three times. The mixture was vibrated in the water bath at 60 °C for certain time (Sample b: 8 h, Sample c: 16 h, Sample d: 24 h). Then, HRP catalyzed silk-grafted-poly(HPMA) (HC-silk-g-PHPMA) sample was obtained and washed with methyl alcohol and water, and finally dried at room temperature under vacuum to a constant weight. 

#### 2.3.2. CuBr-Mediated Grafting of HPMA on Silk Fabric’s Surfaces

The silk-Br macroinitiator (1 g) was immersed into a reaction mixture containing certain HPMA, CuBr/PMDETA(N,N,N′,N″,N″-pentamethyldiethylenetriamine), and 50 mL of deionized water in a 100 mL round-bottom flask ([HPMA] = 0.24 mol/L, [CuBr] = 0.16 mmol/L, n(PMDETA):n(CuBr) = 2:1). After sealing it with a polytetrafluoroethylene three-way stopcock, the flask was evacuated and flushed with nitrogen, which was repeated three times. The mixture was placed in water bath and polymerized under oscillation at 80 °C for 6 h. The sample was rinsed with dilutedhydrochloric acid to remove the blue color caused by CuBr, and then washed with acetylacetone/ethanol (volume ration: 1/5) and water, and dried under a vacuum oven. Thus, CuBr/PMDETA catalyzed silk-grafted-poly(HPMA) (CC-silk-g-PHPMA) sample was obtained.

#### 2.3.3. Grafting Yield Calculation

Grafting yield was calculated as follows:

(1) $$\mathrm{Grafting}\;\mathrm{yield}\;(\%)=\frac{w_{2}-w_{1}}{w_{1}}\times 100$$

in which *w*_1_ and *w*_2_ denote the weight of the control silk and the PHPMA grafted silk fabric, respectively.

### 2.4. Characterization and Measurements

#### 2.4.1. Fourier Transform Infrared (FT-IR) Analysis

The FTIR spectra were recorded using a Nicolet5700 FTIR (Nicolet Co., Madison, WI, USA). The scan range was from 4000 to 400 cm^−1^.

#### 2.4.2. X-ray Diffraction (XRD) Analysis

XRD patterns were obtained at a scanning rate of 1°/min using an X’Pert PRO MPD diffractometer (Holland Panalytical, Almelo, Holland). The voltage and current of the X-ray source were 40 kV and 30 mA, respectively.

#### 2.4.3. X-ray Photoelectron Spectroscopy (XPS) Analysis

X-ray photoelectron spectroscopy (XPS) was carried out on a Thermo ESCALAB 250 X-ray photoelectron spectroscopy(Thermo Fisher Scientific, Waltham, MA, USA) using Al Ka (1486.6 eV) excitatior with pass energy of 20 eV at a reduced power of 150 W. The samples were attached to the spectrometer probe with double-sided adhesive tape, and the X-ray beam was 500 μm.

#### 2.4.4. Thermal Properties

Thermogravimetic analysis (TGA) measurements were performed on a Diamond 5700 thermal analyzer at a heating rate of 10 °C/min with a temperature range from 40 to 600 °C. The open aluminum cell was swept with N_2_ during the analysis.

#### 2.4.5. Scanning Electron Microscopy (SEM) Analysis

Morphology of the silk samples was observed at 3.00 k magnification by a Hitachi TM3030 Desktop SEM (Hitachi TM3030, Hitachi Ltd., Tokyo, Japan) at an acceleration voltage of 3 kV under vacuum condition. The samples were mounted on a conductive adhesive tape and coated with gold before testing.

#### 2.4.6. Crease-Resistant Recovery and Physical Properties Measurement

The wrinkle recovery angle of the fabric was measured according to AATCC66-2003 (Wrinkle Recovery of Woven Fabric: Recovery Angle). Each result was the average of six measurements. The tensile strength of the fabric was tested by an Instron 3365 Universal Testing Machine (IllinoisTool Works Inc., High Wycombe, Buckinghamshire, UK) according to ISO 13934-1-2013. The sample was cut into the size of 30 cm × 5 cm and the average value was obtained after 5 times tests. The whiteness of silk fabric was measured by WSD III whiteness instrument (Wenzhou Darong Textile Instrument Co., Ltd., Wenzhou, China), and the result was the average of eight measurements. Moisture regain (MR) was evaluated in the standard conditions at 20 °C and 65% relative humidity (RH) (ISO 2060: Determination of moisture content and moisture regain of textile-oven-drying method, 1994).MR was calculated according to the following equation:

(2) $$\mathrm{MR}\;(\%)=\frac{m_{1}-m_{0}}{m_{0}}\times 100$$

in which *m*_0_ is weight of dried fabric, and *m*_1_ is weight of moist fabric.

## 3. Results and Discussion

### 3.1. Fourier Transform Infrared (FTIR)Spectra and X-ray Diffraction (XRD) Curves

**Figure 2:**
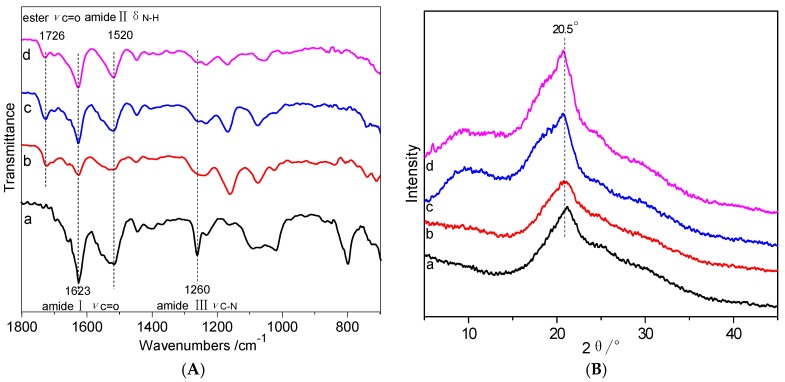
FTIR spectra (**A**) and XRD curves (**B**) of (a) the control silk fabric, (b) 15.62% of HC-silk-g-PHPMA, (c) 29.01% of HC-silk-g-PHPMA, and (d) 38.87% of HC-silk-g-PHPMA.

Figure 2 shows the FTIR spectra and XRD curves of the silk samples before and after grafting. The FTIR spectra (Figure 2A) of the silk all show the characteristic absorption peaks of silk fibroin including amide Iυ_C=O_ 1726 cm^−1^, amide IIδ_N-H_ 1623 cm^−1^, and amide IIIυ_C-N_1260 cm^−1^. Compared with the control silk fabric, additional peak appeared at 1726 cm^−1^ for HC-silk-g-PHPMA, which is characteristic absorption peak of carbonyl stretching vibration of ester, indicating that the HPMA monomers were successfully grafted onto silk fabric.XRD patterns are analyzed as shown in Figure 2B. It could be seen that control silk fabric and the grafted silk fabric all exhibited a major X-ray diffraction peak at 20.5°, which is characteristic peak of silk with highly ordered β-structure [4]. The position and intensity of the major X-ray diffraction peak did not change regardless of the grafting. That is to say, the grafting has no effect on the crystalline region of silk fibers, and it is also reasonable to assume that the grafting is not harsh and causes no damage to the crystalline region of silk fibers.

### 3.2. X-ray Photoelectron Spectroscopy (XPS)

**Figure 3:**
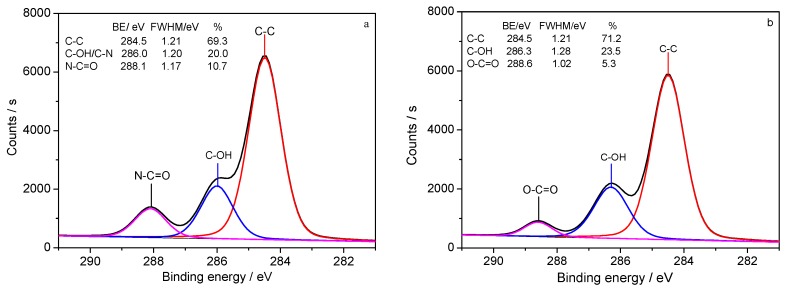
The curve fitting analysis of C1s spectra of (**a**) the control silk fabric and (**b**) 38.87% of HC-silk-g-PHPMA.

The XPS C1s spectra of silk fabric are shown in Figure 3. The C1s spectrum of the control silk fabric contains three distinct peaks at 284.5 (C–C), 286.0 (C–OH/C–N), and 288.1 eV (N–C=O) [22], while the C1s spectrum of HC-silk-g-PHPMA contains three distinct peaks at 284.5 (C–C), 286.3 (C–OH), and 288.6 eV (O–C=O). The C–OH (286.3 eV) and O–C=O (288.6 eV) mainly originated from the hydroxyl and ether of HPMA. Table 1 liststhe carbon-to-nitrogen (C/N) ratio of silk fabric, which increased after grafting with HPMA. Less N element content was detected for the grafted silk sample compared with the control silk fabric. The reason was that the surface of the grafted silk fabric was covered by PHPMA polymer, which mainly contains C, O, and H elements. N element was weakened after grafting, further indicating that monomers were successfully grafted onto the silk fabric using HRP biocatalytic ATRP method.

### 3.3. Thermal Properties

**Figure 4:**
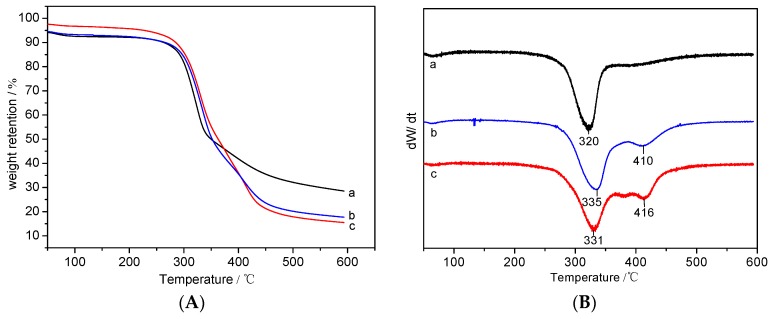
TG curves (**A**) and DTG curves (**B**) of (a) the control silk fabric, (b) 29.01% of HC-silk-g-PHPMA, and (c) 38.87% of HC-silk-g-PHPMA.

Figure 4 shows the TG (A) and DTG (B) curves of the silk fabric. It can be seen from Figure 4A that the evaporation of the absorbed moisture caused slight weight loss of silk fabric when temperature was less than 200 °C. The weight loss ratio of the control silk fabric was 32.5% when the weight loss rate reached its highest point at 320 °C, which could be attributed to the decomposition of silk fabric into small molecules including CO_2_ and H_2_O. The decomposition process of the grafted silk fabric contained two stages, and the weight loss rate reached its highest point at the first stage 331 °C and second stage 410 °C (for 29.01% of HC-silk-g-PHPMA), and 335 and 416 °C (for 38.87% of HC-silk-g-PHPMA), respectively. From Figure 4B, the peaks at 331 (b, DTG) and 335 °C (c, DTG) of HC-silk-g-PHPMA in the first stage were caused by the decomposition of silk fibroin, which corresponded to the peak at 320 °C (a, DTG) of control silk fabric. At the second stage, the grafted silk fabric had additional peaks at 410 and 416 °C, which can be explained by the decomposition of poly(HPMA) [23]. When heated up to 600 °C, the final residual of the control silk fabric (28.5%) was more than that of the grafted silk fabric (15.4%), which may be due to the decomposition of the poly-(HPMA) into small molecules such as CO_2_ and H_2_O. This phenomenon further indicated the monomers were successfully grafted onto the silk fabric using HRP-catalyzed atom transfer radical polymerization method.

### 3.4. Scanning Electron Microscopy (SEM)

**Figure 5:**
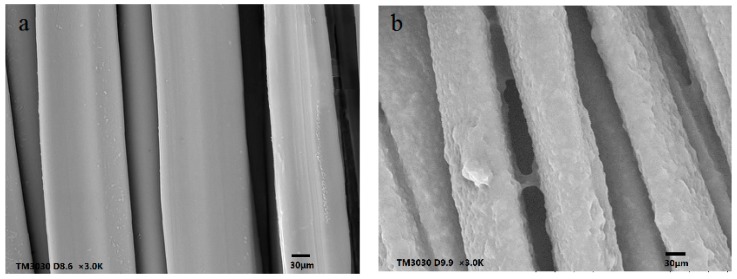

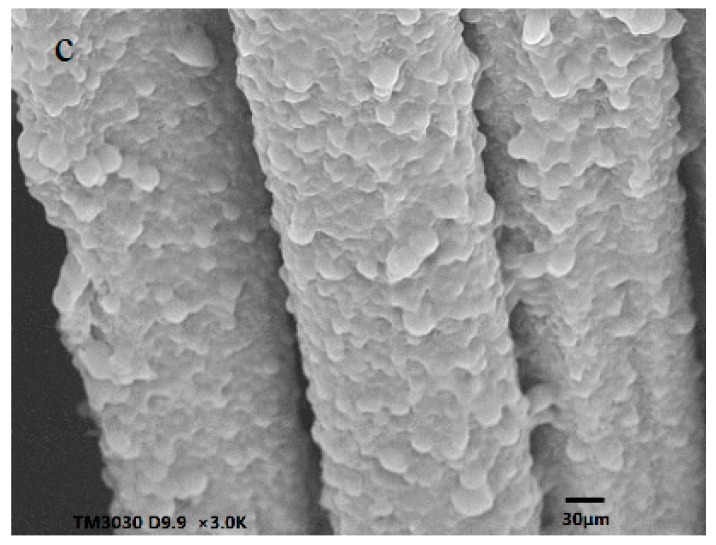
SEM images of (**a**) the control silk fabric, (**b**) 29.01% the HC-silk-g-PHPMA, and (**c**) 38.87% the HC-silk-g-PHPMA.

Figure 5 shows the morphology of silk fabrics. The control silk fabric had a smooth and uniform appearance in portrait orientation. The surfaces of the grafted silk fabric were covered with PHPMA and became tough. Moreover, the surfaces of the silk fabric were rougher as the grafting yield of the silk fabric became higher.

### 3.5. Crease-Resistant Recovery

Compared with the control silk fabric, the crease-resistant recovery properties of the silk fabric grafted with HPMA using HRP biocatalys is and metal complex catalysis method were both improved (Table 2). With the increase of grafting yield, the dry crease recovery angle (DCRA) and wet crease recovery angle (WCRA) of HC-silk-g-PHPMA increased up to 18.41% and 51.56%, respectively. This can be attributed to two reasons: (1) The copolymerization reaction of monomer and silk fabric occurred in amorphous area of silk fiber, which weaken the hydrogen bonding in amorphous area and decreased the creep deformation and permanent deformation caused by breaking up of hydrogen bonding; (2) The grafting copolymerization in amorphous area limited relative slippage of silk macromolecules. Additionally, the WCRA of grafted silk fabric increased higher than that of the DRCA, which is favorable for silk fabric easy to wrinkle at wet state. This result can be attributed to the occurrence of grafting under wet state. The DCRA and WCRA of CC-silk-g-PHPMA also have the same increase trend with HC-silk-g-PHPMA. 

### 3.6. Physical Properties

Compared with control silk fabric, HC-silk-g-PHPMA and CC-silk-g-PHPMA were somewhat damaged in the whiteness index and breaking strength (Table 3). For HC-silk-g-PHPMA, the inactive enzymes adhered to the surface of silk fabrics reduced the whiteness of silk fabrics. For CC-silk-g-PHPMA, the colored CuBr stained the silk fabric and also caused the decrease of whiteness. The mechanical properties of fibers partly depend on the orientation of macromolecule. After grafting with HPMA, the orientation of macromolecule of silk fiber was changed, and the breaking strength decreased. The balance moisture regain of grafted silk fabrics reduced because the hydrophilic groups of silk fabric surface were covered by the polymers. The physical properties of HC-silk-g-PHPMA and CC-silk-g-PHPMA were basically the same, but the advantage of HC-silk-g-PHPMA is the usage of HRP biocatalyst.

## 4. Conclusions

In conclusion, silk fabric was successfully grafted with 2-hydroxypropyl methacrylate (HPMA) using the HRP-mediated ATRP method. The structure of control silk and grafted silk fabric was characterized by Fourier transform infrared, XRD, XPS, TG, and SEM. The results indicated HPMA was grafted onto the surface of silk fabric, and the copolymerization occurred in the amorphous region of silk fabric. Compared with the control sample, the grafted silk fabric showed greatimprovement in the crease-resistant recovery property, especially in the wet crease recovery angle. However, the whiteness, breaking strength, and moisture regain of grafted silk fabric decreased.In comparison with HC-silk-g-PHPMA and CC-silk-g-PHPMA, its properties were nearly the same, and the biocatalyst HRP was applied in the preparation of HC-silk-g-PHPMA. Consequently, this work provides a biocatalyzed ATRP method with which to obtain functionalsilk fabric, which might realize the potential application of the ATRP grafting method in textile and medical materialsmodification.

## Figures and Tables

**Table 1:** The element weight percent of (a) the control silk fabric and (b) 38.87% ofHC-silk-g-PHPMA.

Silk Sample	Element Content/%			C/N
	C	O	N	
Control	69.98	24.08	5.94	11.78
silk-g-PHPMA	71.20	26.31	2.49	28.59

**Table 2:** The CRA of silk fabric with different grafting yield under dry and wet conditions.

Grafting Samples	Grafting Yield/%	DCRA/°	WCRA/°
	0	201	128
HC-silk-g-PHPMA	15.62	220	168
	29.01	233	180
	38.87	238	194
CC-silk-g-PHPMA	37.82	229	195

**Table 3:** Whiteness, breaking strength, and moisture regain of silk fabrics.

Grafting Samples	Grafting Yield/%	Whiteness/%	Breaking Strength/N	Moisture Regain/%
	0	79.02	479.74	8.45
HC-silk-g-PHPMA	15.62	75.68	428.25	8.03
	29.01	74.26	415.36	7.96
	38.87	70.39	391.59	7.78
CC-silk-g-PHPMA	37.82	70.23	396.86	7.98
